# LINAC-based SBRT in treating early-stage NSCLC patients—single institution experience and survival data analysis

**DOI:** 10.3389/pore.2024.1611589

**Published:** 2024-02-13

**Authors:** Árpád Kovács, Krisztina Trási, Márton Barabás, Kristóf Gál, Emese Csiki, Dávid Sipos, Judit Papp, Mihály Simon

**Affiliations:** ^1^ Department of Oncoradiology, Faculty of Medicine, University of Debrecen, Debrecen, Hungary; ^2^ Doctoral School of Health Sciences, Faculty of Health Sciences, University of Pécs, Pécs, Hungary

**Keywords:** VATS, NSCLC, SBRT, LINAC-based SBRT, early stage

## Abstract

**Aim:** This single institute prospective study aimed to evaluate the feasibility of LINAC-based stereotactic body radiotherapy (SBRT) in treating patients with early-stage non-small cell lung cancer (NSLSC). We focused on the survival data with the local and distant control profiles and the cancer- and non-cancer-specific survival. Treatment-related side effects were also collected and analyzed.

**Methods:** Patients with early-stage NSCLC between January 2018 and October 2021 were included in our prospective study; a total of 77 patients receiving LINAC-based SBRT were analyzed. All patients had pretreatment multidisciplinary tumor board decisions on SBRT. The average patient age was 68.8 years (median: 70 years, range: 52–82); 70 patients were in ECOG 0 status (91%), while seven patients were in ECOG 1-2 status (9%). 52% of the patients (40) had histologically verified NSCLC, and the other 48% were verified based on PETCT results. We applied the SBRT scheme 8 x 7.5 Gy for central tumors (74%) or 4 x 12 Gy for peripheral tumors (26%).

**Results:** The mean follow-up time was 25.4 months (median 23, range 18–50). The Kaplan-Meier estimation for overall survival in patients receiving LINAC-based SBRT was 41.67 months. Of the 77 patients treated by SBRT, death was reported for 17 patients (9 cases cancer-specific, 8 cases non-cancer specific reason). The mean local tumor control was 34.25 months (range 8.4–41), and the mean systemic control was 24.24 months (range 7–25). During the treatments, no Grade I-II were reported; in 30 cases, Grade I non-symptomatic treatment-related lung fibrosis and two asymptomatic rib fractures were reported.

**Conclusion:** In the treatment of early-stage NSCLC, LINAC-based SBRT can be a feasible alternative to surgery. Although we reported worse OS data in our patient cohort compared to the literature, the higher older average age and the initial worse general condition (ECOG1-2) in our patient cohort appear to be the reason for this difference. With the comparable local control and survival data and the favorable side effect profile, SBRT might be preferable over surgery in selected cases.

## Introduction

Lung cancer in Europe represents a leading cause of cancer cases, with more than 312,000 newly diagnosed cases per year. Hungary is the leading European country in the incidence of lung cancer and has the highest mortality rate [1, 2]. Approximately 85% of all lung cancer incidences are non-small cell lung cancer (NSCLC) [3]. The gold standard curative protocol is surgery that mainly aims to reduce the disease progression, relieve the symptoms, and increase the overall survival (OS) if possible [4]. Many patients, however, are unable to tolerate thoracotomy due to comorbidities or personal preference. [5] The video-assisted thoracoscopic surgery (VATS) method was chosen as the treatment choice since it was reported to decrease the risk of complication after treatment and a higher 5-year survival rate than the open lobectomy method [5, 6]. Nevertheless, in some cases where resection is not possible due to the tumor location, functional status of the lung, or inoperable patients [7, 8], another treatment method should be evaluated and assessed.

LINAC-based stereotactic body radiation therapy (SBRT), which is an alternative to VATS, was found to be a choice of treatment, especially for elderly patients and those patients with more than one known disease [5]. This method was comparable to the VATS in previous clinical reports [5, 9–11].

SBRT is a state-of-the-art treatment method that uses radiation therapy to deliver high-dose ablative doses to tumors [11]. This method allows for successful tumor ablation with relatively high tumor control probability while keeping the surrounding tissues intact [7, 12]. Furthermore, this technology’s use and continuous improvement can improve results in potentially operable cases. Previously, limited studies have been compared between (SBRT) and (VATS) in terms of overall survival (OS), cancer-specific survival (CSS), loco-regional control (LCC), and disease-free survival (DFS). Therefore, this single institute prospective study aims to evaluate LINAC-based SBRT for NSCLC patients as 5 years of follow-up experience at Debrecen University regarding OS, CSS, LCC, and side effect profile.

## Material and methods

### Study population

This was a prospective mono-institutional study; the consent form was obtained from each patient. Patient data were collected and processed with the ethical permission of the Regional Research Ethics Committee. Demographic variables obtained from the electronic file database Clinic Center of the University of Debrecen (Debrecen, Hungary), called UD-MED, included age, gender, forced expiratory volume in 1 s to forced vital capacity ratio (FEV1/FVC%), and FEV1% predicted before treatment. All patients underwent pretreatment multidisciplinary tumor board before starting the SBRT at the Oncoradiology Clinic of the University of Debrecen (Debrecen, Hungary).

The average age was 68.8 years (median: 70 years, range: 52–82); 67 patients were in ECOG 0 status (87%), while ten patients were in ECOG 1-2 status (13%). 52% of the patients (40) had histologically verified NSCLC, and 48% were confirmed based on PET-CT with high FDG SUV (over the SUV value of 2.8). The mean FEV1 value was 1.06 (L), the mean FEV1 42%, the mean FVC 2.14 (L), and the mean FVC 62.69%.

### Imaging

Chest CT examination is fundamental in tumor diagnostics and staging; a contrast-enhanced chest CT scan was used in all cases as a part of staging. Determining the patient’s respiratory function capacity was also crucial from the point of view of the operation and the execution of the radiation treatment. Before treatment, a bronchoscopy was performed in all cases. In cases where the bronchoscopy could not give proper histological information, a CT-guided needle biopsy was conducted where a better visualization of the tumor’s position was obtained when the tumor was smaller than 2 cm and when complications were more avoidable with such an examination. To decide oncological operability, enlarged lymph nodes detected on CT or PET-CT were valid only in conjunction with a positive histological examination. Suspicious patterns examined with CT can be supplemented with an FDG-PET examination, and in the case of non-small cell lung cancer, increased FDG uptake is observed. All patients receiving SBRT in our patient cohort had pretreatment FDG-PET scans within 2 weeks before the start of the treatment. A brain MRI was performed in all cases as a part of the staging.

### Treatment procedures

For the 4D CT-based SBRT procedures, we used ELEKTA VERSA HD units with individual vacuum fixation systems and online 4D CBCT verification for each patient. Planning 4D CT was performed in the treatment position, with a slice thickness of 3 mm. No abdominal compression was applied during the process. Radiotherapy contouring and planning followed the department clinical protocol using the Pinnacle (Phillips, Netherlands) planning system (System version 16.2). To determine the exact gross tumor volume (GTV) and biological target volume (BTV), 4D planning CT-fused with FDG PET scans was used. Besides, the GTV and BTV internal target volume (ITV) was defined, using 4D CT information, to cover tumor movements. An additional 3–5 mm margin was used to generate the PTV. The mandatory OARs in planning were the lungs, heart, spinal cord, trachea, esophagus, chest wall, and great vessels per protocol. We applied the SBRT scheme of 8 × 7.5 Gy for central tumors (74%) or 4 × 12 Gy for peripheral tumors (26%). The treatments were delivered every other day (48-h shifts), with daily 4D CBCT verification and correction, if needed.

### Data collection

Patients were followed up as follows: every 3 months for 2 years, every 6 months for another 3 years, and then annually. A medical history, physical examination, and chest CT were performed during the follow-up.

The primary endpoint of the study was local control (LC). We also examined systemic control, cancer-specific survival (CSS), and non-cancer-specific survival (NCSS). The analysis also focused on overall survival (OS) and treatment-related toxicity. OS was defined as the interval from the treatment date to any death or the last follow-up.

### Statistical analysis

We used UD-MED, MEDSOL, and the Electronic Health Service Space (EESZT) for clinical data collection and analysis. The statistical analysis was performed with in-house-built Python scripts using the lifelines (v0.27.8) package [13].

**FIGURE 1:**
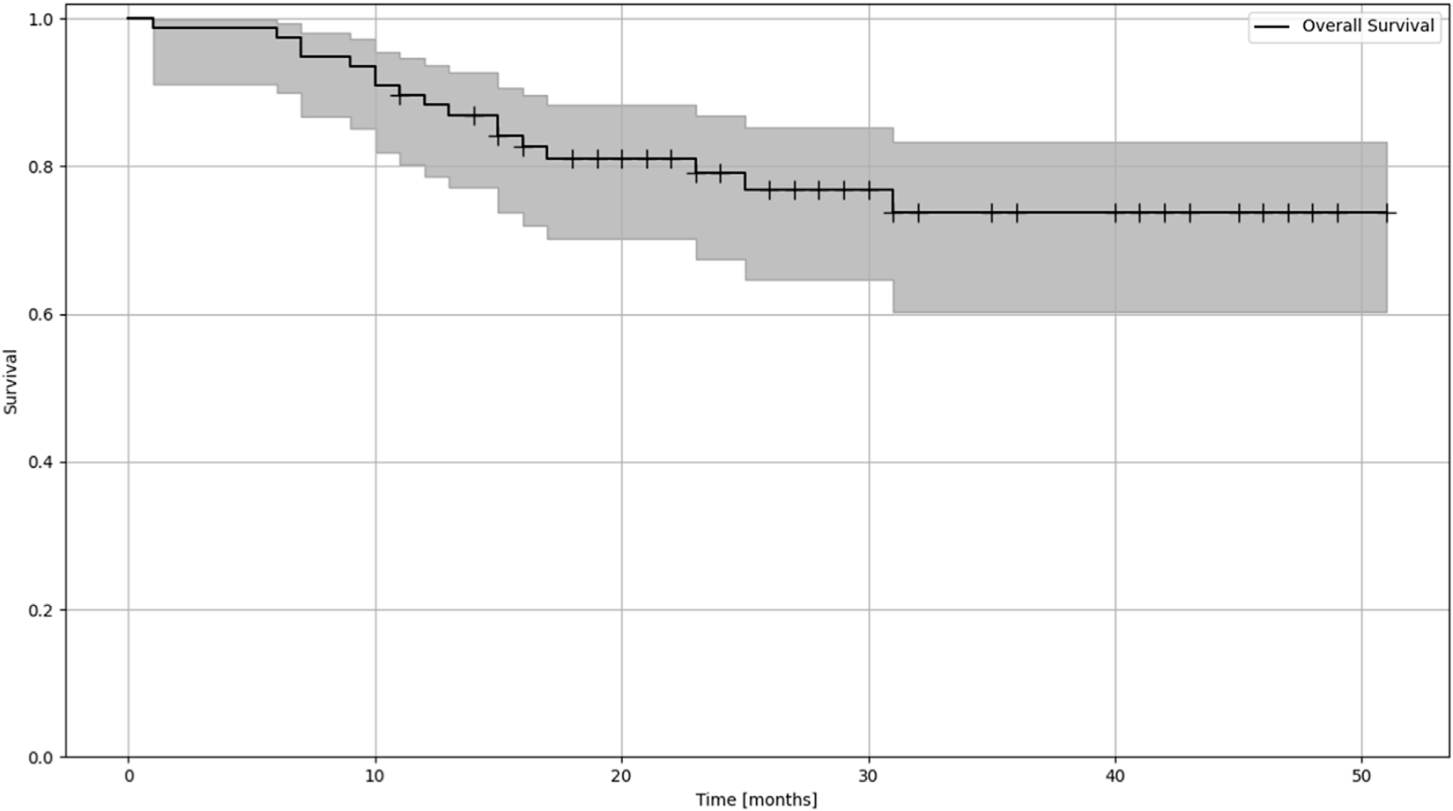
Kaplan-Meier estimation of OS (overall survival) of patients treated with lung SBRT.

**FIGURE 2:**
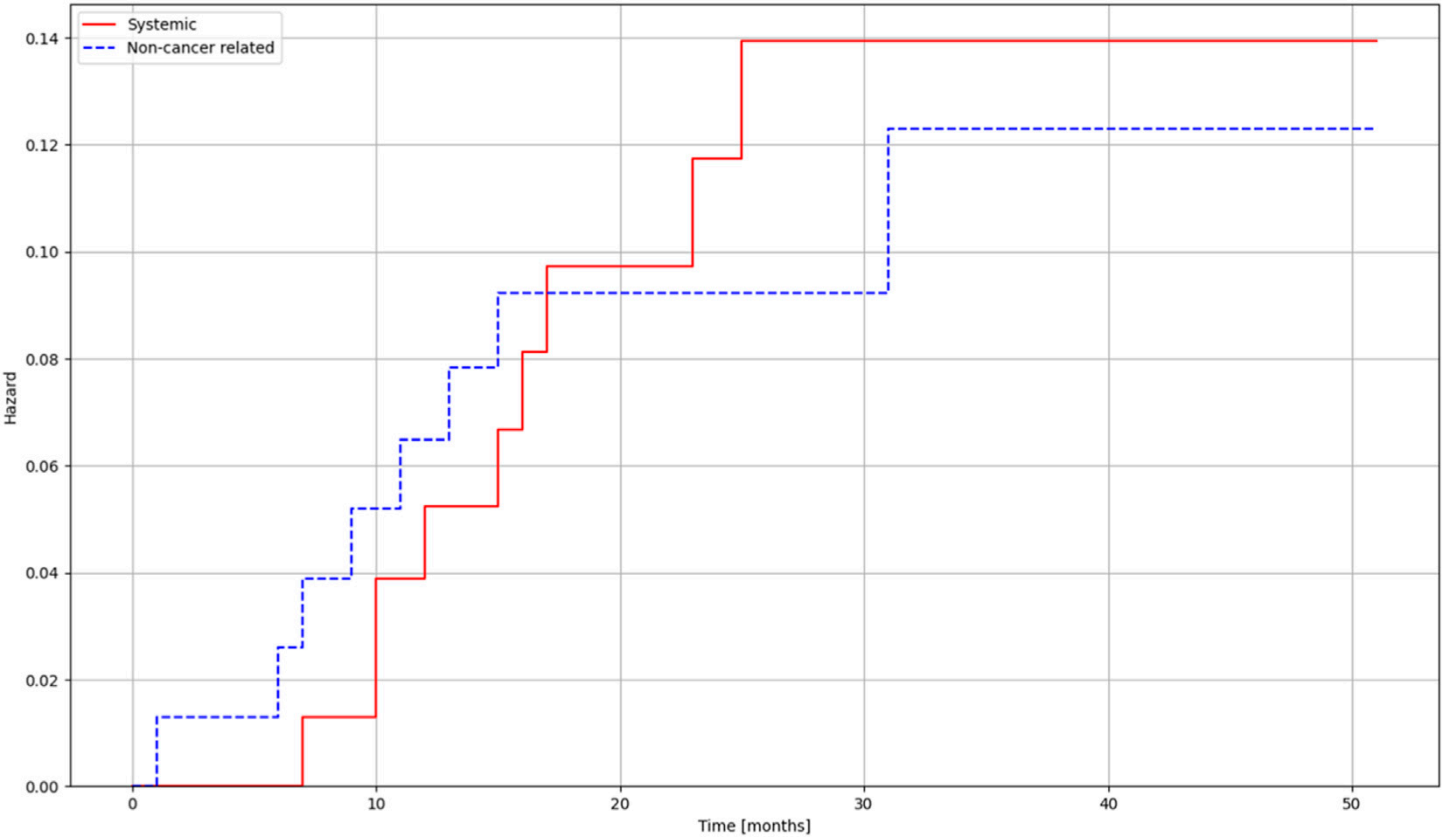
Risk estimates for deaths related to systemic progression and non-cancer-related deaths for patients treated with lung SBRT.

Overall survival was estimated with the Kaplan Meier method (Figure 1). Risk estimation was performed using the Aalen-Johansen estimator to be able to assess risk in the different groups of patinets (Figure 2, Table 1).

**TABLE 1:** Aalen-Johansen risk estimate for cumulative risk of patients.

	Times [months]					
	0	10	20	30	40	50
At risk	77	72	46	25	12	3
Censored	0	0	17	36	48	57
Events	0	5	14	16	17	17

## Results

The mean follow-up time was 25.4 months (median 23, range 18–50). The Kaplan-Meier estimation for overall survival in patients receiving LINAC-based SBRT was 41.67 months. Of the 77 patients treated by SBRT, death was reported for 17 patients (9 cases cancer-specific, 8 cases non-cancer specific reason). The mean local tumor control was 34.25 months (range 8.4–41), and the mean systemic control was 24.24 months (range 7–25) During the treatments, no Grade I-II side effect were reported; in 30 cases, Grade I non-symptomatic treatment-related lung fibrosis and two asymptomatic rib fractures were reported.

## Discussion

The gold standard treatment option for early-stage NSCLC patients is still surgery, considered the first treatment of choice [14–16]. The state of art surgery is usually done with VATS to reduce patient encumbrance [17–19]. Considering the Overall Survival, Loco-regional Control, and Systemic Control data of the previously reported retrospective studies, SBRT is a full-fledged alternative to surgery for early-stage NSCLC patients [5, 20–22]. Besides the comparable local control and survival data, the main advantage of the SBRT is the favorable side effect profile and excellent tolerability, even in comorbid elderly patients [23]. Using SBRT also offers lower post‐treatment mortality [24]. In the literature, only a few studies focus on comparing SBRT and surgery because the comparison is made difficult by the patients’ different average ages and health statuses [5, 10–12, 25]. In our patient cohort, the higher average patient age, worse general condition, and initial respiratory functions are all reflected in the general patient selection process in the clinical decisions; the younger patients in good general condition are more frequently referred to surgery.

As in the previous studies, Dong et al., in their analysis of several studies, found that the results of SBRT were comparable to those of VATS. Thus, they reported that OS was comparable between the two groups with a statistically significant difference. They also reported comparable outcomes with no significant differences in terms of loco-regional failure, with 3- and 5-year rates of loco-regional failure for radiotherapy and surgery being 93.5% and 93.5% and 94.0% and 85.9%, respectively; furthermore, they reported that distal failure was comparable for both groups with no statistical significance between the groups [5].

In our prospective study, the SBRT-related loco-regional and systemic control results are comparable to the previously reported conventional surgery results in the literature [5, 7]. In the SBRT cohort, the hazard of death due to systemic progression barely exceeds the risk of dying from non-cancer-related reasons. We recorded no deaths due to local progression. For systemic control, we also examined the lymph node and distant metastases; SBRT showed promising results in terms of non-tumor-specific survival. The scope of SBRT indications should be expanded in the future, and further studies with more cases should be considered. Currently, the gold standard therapy of choice is still surgery [14, 16, 25], the advantage of which is the possibility of histological sampling.

It is important to note that in our prospective data analysis, some data were worse for patients who underwent SBRT, as reported in the literature; this can be explained by the fact that in our SBRT group, there were medically inoperable patients with worse respiratory function and with many comorbidities. Pulmonary function values were available before and after SBRT in some patients, and we observed improvement in FEV1 and FVC. The difference between recurrence and fibrosis can be difficult during follow-up, so monitoring the patient with PET-CT is essential. Another difficulty is the separation of metastases and secondary lung tumors.

Our patient cohort also noticed no acute side effects during SBRT. No late severe side effects were also described during oncological follow-up. This study aims to help clinicians the find the proper treatment of patients with early-stage NSCLC. The findings provided valuable information in answering these and other unresolved questions regarding SBRT.

## Conclusion

In the treatment of early-stage NSCLC, LINAC-based SBRT can be a feasible alternative to surgery. We report moderately worse OS data in our patient cohort compared to the literature [5]. However, the difference in average age and the initial worse general condition (ECOG1-2) of our patient cohort can be an underlying reason. With the comparable local control and survival data and the favorable side effect profile, SBRT might be preferable over surgery in selected cases.

## Data Availability

The original contributions presented in the study are included in the article materials, further inquiries can be directed to the corresponding author. All the datasets are available to access.

## Abbreviations

NSCLS, Non-small cell lung cancer; VATS, video-assisted thoracoscopic surgery; SBRT, Stereotactic body radiation therapy; LC, Local control; OS, Overall survival; CSS, cancer-specific survival; LCC, locoregional control; DFS, disease-free survival.
